# 2D and 3D convolutional neural networks for outcome modelling of locally advanced head and neck squamous cell carcinoma

**DOI:** 10.1038/s41598-020-70542-9

**Published:** 2020-09-24

**Authors:** Sebastian Starke, Stefan Leger, Alex Zwanenburg, Karoline Leger, Fabian Lohaus, Annett Linge, Andreas Schreiber, Goda Kalinauskaite, Inge Tinhofer, Nika Guberina, Maja Guberina, Panagiotis Balermpas, Jens von der Grün, Ute Ganswindt, Claus Belka, Jan C. Peeken, Stephanie E. Combs, Simon Boeke, Daniel Zips, Christian Richter, Esther G. C. Troost, Mechthild Krause, Michael Baumann, Steffen Löck

**Affiliations:** 1 Helmholtz-Zentrum Dresden - Rossendorf, Department of Information Services and Computing, Dresden, Germany; 2 OncoRay - National Center for Radiation Research in Oncology, Faculty of Medicine and University Hospital Carl Gustav Carus, Technische Universität Dresden, Helmholtz-Zentrum Dresden - Rossendorf, Dresden, Germany; 3 German Cancer Research Center (DKFZ), Heidelberg and German Cancer Consortium (DKTK) partner site Dresden, Dresden, Germany; 4 National Center for Tumor Diseases (NCT), Partner Site Dresden, Germany; 5 Department of Radiotherapy and Radiation Oncology, Faculty of Medicine and University Hospital Carl Gustav Carus, Technische Universität Dresden, Dresden, Germany; 6 Department of Radiotherapy, Hospital Dresden-Friedrichstadt, Dresden, Germany; 7 German Cancer Research Center (DKFZ), Heidelberg and German Cancer Consortium (DKTK) partner site Berlin, Berlin, Germany; 8 Department of Radiooncology and Radiotherapy, Charité University Hospital, Berlin, Germany; 9 German Cancer Research Center (DKFZ), Heidelberg and German Cancer Consortium (DKTK) partner site Essen, Essen, Germany; 10 Department of Radiotherapy, Medical Faculty, University of Duisburg-Essen, Essen, Germany; 11 German Cancer Research Center (DKFZ), Heidelberg and German Cancer Consortium (DKTK) partner site Frankfurt, Frankfurt, Germany; 12 Department of Radiotherapy and Oncology, Goethe-University Frankfurt, Frankfurt, Germany; 13 German Cancer Research Center (DKFZ), Heidelberg and German Cancer Consortium (DKTK) partner site Munich, Munich, Germany; 14 Department of Radiation Oncology, Ludwig-Maximilians-Universität, Munich, Germany; 15 Clinical Cooperation Group, Personalized Radiotherapy in Head and Neck Cancer, Helmholtz Zentrum, Munich, Germany; 16 Department of Radiation Oncology, Medical University of Innsbruck, Anichstraße 35, 6020 Innsbruck, Austria; 17 Department of Radiation Oncology, Technische Universität München, Munich, Germany; 18 Institute of Radiation Medicine (IRM), Helmholtz Zentrum München, Neuherberg, Germany; 19 German Cancer Research Center (DKFZ), Heidelberg and German Cancer Consortium (DKTK) partner site Tübingen, Tübingen, Germany; 20 Department of Radiation Oncology, Faculty of Medicine and University Hospital Tübingen, Eberhard Karls Universität Tübingen, Tübingen, Germany; 21 Helmholtz-Zentrum Dresden - Rossendorf, Institute of Radiooncology – OncoRay, Dresden, Germany; 22 German Cancer Research Center (DKFZ), Heidelberg, Germany

**Keywords:** Predictive markers, Cancer imaging, Head and neck cancer

## Abstract

For treatment individualisation of patients with locally advanced head and neck squamous cell carcinoma (HNSCC) treated with primary radiochemotherapy, we explored the capabilities of different deep learning approaches for predicting loco-regional tumour control (LRC) from treatment-planning computed tomography images. Based on multicentre cohorts for exploration (206 patients) and independent validation (85 patients), multiple deep learning strategies including training of 3D- and 2D-convolutional neural networks (CNN) from scratch, transfer learning and extraction of deep autoencoder features were assessed and compared to a clinical model. Analyses were based on Cox proportional hazards regression and model performances were assessed by the concordance index (C-index) and the model’s ability to stratify patients based on predicted hazards of LRC. Among all models, an ensemble of 3D-CNNs achieved the best performance (C-index 0.31) with a significant association to LRC on the independent validation cohort. It performed better than the clinical model including the tumour volume (C-index 0.39). Significant differences in LRC were observed between patient groups at low or high risk of tumour recurrence as predicted by the model ($p=0.001$). This 3D-CNN ensemble will be further evaluated in a currently ongoing prospective validation study once follow-up is complete.

## Introduction

Treatment individualisation is a central objective for the improvement of radiotherapy outcomes^1^. In particular, patients diagnosed with locally advanced head and neck squamous cell carcinoma (HNSCC) might benefit from individualised treatment, since five-year overall survival probability after primary radiochemotherapy is only approx. 50%^2^. Subgroups of patients may be identified that are currently under- or overtreated and might benefit from e.g. escalated or de-escalated dose prescriptions. Individualisation of treatment may be based on statistical survival models that predict endpoints such as overall survival or loco-regional tumour control (LRC). Survival models are able to analyse time-to-event data which frequently contain censored observations. The prognostic value of these models is based on biomarkers that are able to stratify patients into groups at different risk of treatment failure. Such biomarkers may result from clinical or tumour-related features such as age, gender or tumour stage, molecular analyses of tumour biopsies such as human papillomavirus (HPV) status or gene signatures, dosimetric information or clinical imaging data from computed tomography (CT), magnetic resonance imaging (MRI), positron emission tomography (PET) scans or combinations thereof^3–13^.

Imaging data are considered a valuable source of information for tailoring individual treatment, due to their non-invasiveness, repeatability and their ability to represent the entire tumour. Numerous radiomics models, in which traditional machine-learning (ML) methods were applied on hundreds to thousands of pre-defined and handcrafted image features, have been developed^14–18^, but have not yet surpassed the threshold for clinical acceptance and applicability^19^. Recently, Ger et al.^20^ found that radiomics features of CT and PET scans failed to improve upon clinical risk models in a large head and neck cancer dataset. With the recent advances that deep convolutional neural networks (CNNs) have brought to the fields of natural and medical image analysis, there is hope to elevate model performance for radiotherapy outcome modelling, as well. This is mostly due to the fact that CNNs are able to automatically learn abstract feature representations of the input data during training. However, so far most applications of deep learning to medical images revolve around tasks for segmentation^21^ or classification^22–24^. The same holds true for the field of radiotherapy, where most applications of deep learning focus on segmentation, computer-aided detection or motion management^25^. Only few attempts have been published to combine deep learning on medical imaging data and survival analysis^26–28^.

The Cox proportional hazards model (CPHM) is a clinically established survival model. It is often used because it allows to exclusively model effects of patient covariates on individual event times without making distributional assumptions. Previously, Katzman et al.^29^ demonstrated the benefit of combining multi-layer perceptrons with the CPHM for modelling of nonlinear feature interactions, while Ching et al.^30^ applied this approach to ten cancer-related datasets of high throughput transcriptomics data. Additionally, the CPHM and CNNs were combined to build risk models based on pathological histology images for lung cancer and glioblastoma patients, respectively^26,27^, whereas Haarburger et al.^28^ applied a similar idea to CT scans of lung cancer patients.

In this manuscript, we developed and independently validated three deep learning approaches to predict LRC from treatment-planning CT images of patients with locally advanced HNSCC treated by primary radiochemotherapy. (i) We developed a CPHM based only on clinical parameters to provide a baseline model. Subsequently, we investigated different deep learning approaches to the CPHM: (ii) we trained 3D- and 2D-CNNs from scratch, (iii) we applied a transfer learning strategy by fine-tuning pre-trained networks on our dataset and (iv) we used deep features^31–33^ generated by a trained autoencoder^34^.

## Methods

### Patient cohort

A multicentre retrospective cohort consisting of 291 patients with locally advanced HNSCC was collected and divided into an exploratory and an independent validation cohort (206 and 85 patients, respectively). Allocation of patients was based on the different included studies. 149 of the 206 patients of the exploratory cohort were treated in one of the six partner sites of the German Cancer Consortium Radiation Oncology Group (DKTK-ROG) between 2005 and 2011^7^. The remaining 57 patients were treated at the University Hospital Dresden (UKD, Germany) between 1999 and 2006^35^. 51 of the 85 patients of the independent validation cohort were treated within a prospective clinical trial (NCT00180180) at the UKD between 2006 and 2012^3, 9^. 20 additional patients were treated at the UKD and the Radiotherapy Center Dresden-Friedrichstadt between 2005 and 2009 and the remaining 14 patients were treated in Tübingen between 2008 and 2013^36^.

All patients received a CT scan for treatment-planning and were treated by primary radiochemotherapy. Inclusion criteria have previously been described^3,7,9,35^. Ethical approval for the multicentre retrospective analyses was obtained from the Ethics Committee at the Technische Universität Dresden, Germany (EK177042017)^17^. All analyses were carried out in accordance with the relevant guidelines and regulations. Informed consent was obtained from all patients. CT scans were provided as DICOM files with contours of the primary tumour manually delineated and reviewed by experienced radiation oncologists (F.L., K.L., E.G.C.T.). Patient characteristics are summarised in Table 1.

The primary endpoint of this study was LRC, which was defined as the time between the start of radiochemotherapy and local or regional tumour recurrence. For patients with observed loco-regional recurrence, the event time was accompanied by an event indicator variable of 1, whereas for patients without an observed event, the last follow-up time was used together with an event indicator variable of 0.

**Table 1:** Patient characteristics of the exploratory and independent validation cohort: *p* values were obtained by using two-sided Mann–Whitney U-tests for continuous variables and $\chi ^{2}$ homogeneity tests for categorical variables.

Variable	Exploratory cohort (n = 206)		Independent validation cohort (n = 85)		*p* value
	Median	(Range)	Median	(Range)	
Follow up time of patients alive (months)	52.62	(4.27–131.91)	42.55	(7.85–107.27)	0.72
Age (years)	59.00	(39.20–84.50)	55.00	(37.00–76.00)	0.023
Primary tumour volume (${\hbox {cm}}^{3}$)	29.13	(4.52–321.74)	40.62	(2.70–239.07)	0.039

	Number of patients	(%)	Number of patients	(%)	
Gender male/female	174/32	(84/16)	74/11	(87/13)	0.70
cT-stage T1/T2/T3/T4	2/23/51/130	(1/11/25/63)	2/9/30/44	(2/11/35/52)	0.21
cN-stage N0/N1/N2/N3/unknown	30/7/154/15/0	(15/3/75/7/0)	9/8/64/3/1	(11/9/75/4/1)	0.097
UICC-stage I/II/III/IV	0/0/15/191	(0/0/7/93)	1/2/9/73	(1/2/11/86)	0.039
Tumour site oropharynx/oral cavity/hypopharynx/larynx	93/51/62/0	(45/25/30/0)	29/23/28/5	(34/27/33/6)	0.003
p16 status negative/positive/unknown	148/28/30	(72/13/15)	52/5/28	(61/6/33)	0.26
Pathological grading 0/1/2/3/unknown	1/6/131/61/7	(1/3/63/30/3)	0/0/43/35/7	(0/0/51/41/8)	0.071
Smoking status no/yes/unknown	41/163/2	(20/79/1)	13/51/21	(15/60/25)	1.00
Alcohol consumption no/yes/unknown	62/85/59	(30/41/29)	23/25/37	(27/29/44)	0.60
Loco-regional tumour recurrence	84	(41)	28	(33)	0.26

### Design of the analysis

**Figure 1:**
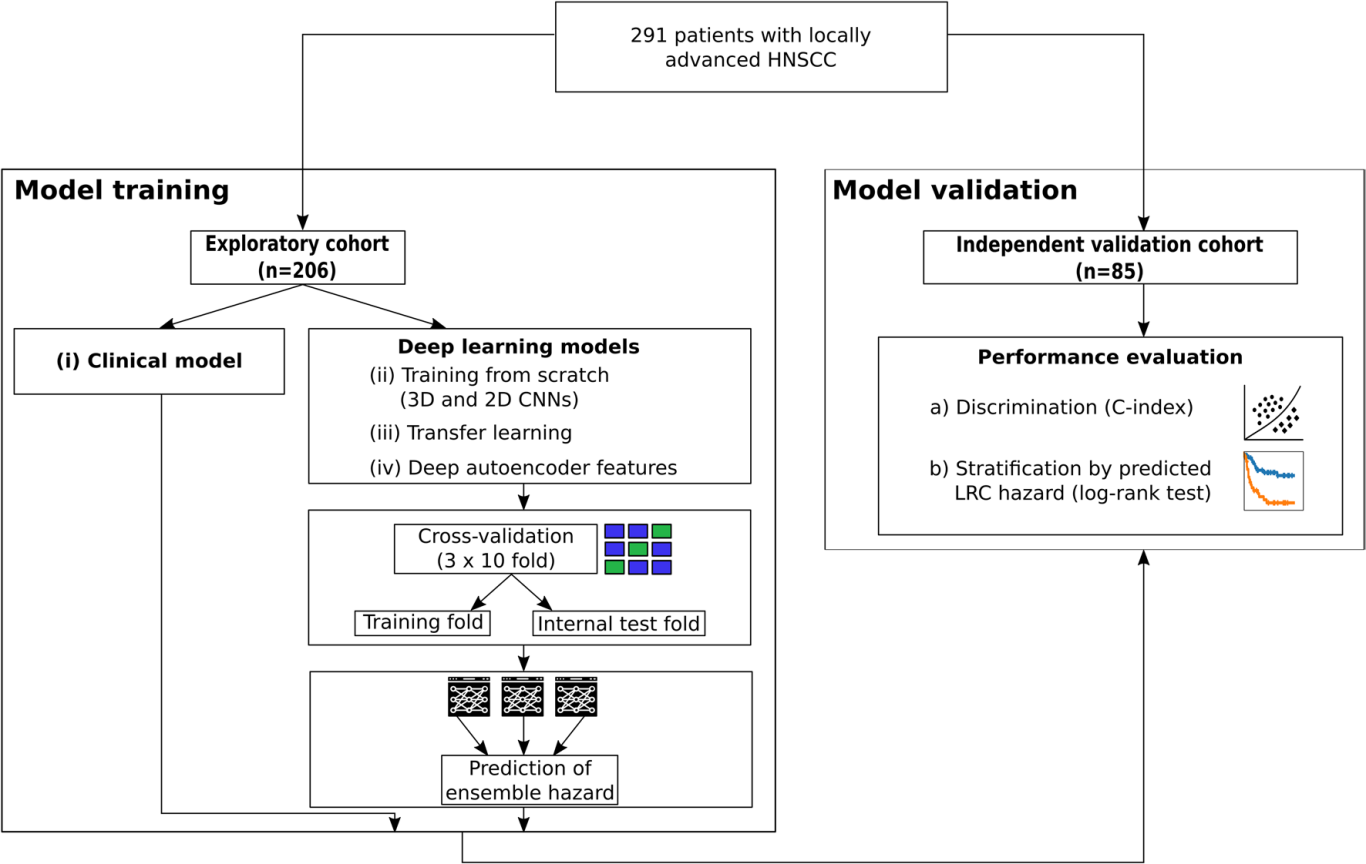
Design of the analysis. (i) To provide baseline results, a clinical Cox proportional hazards model (CPHM) was trained on the exploratory cohort and evaluated on the independent validation cohort. (ii)–(iv) Three deep learning approaches were evaluated by training convolutional neural networks in a cross-validation approach. Subsequently, for each approach ensembles were constructed from the models obtained during cross-validation and their performance was evaluated on the independent validation cohort.

In our analyses we applied the CPHM, which is a regression model commonly used in survival analysis for assessing endpoints like overall survival, progression-free survival or, as in our case, LRC. It is able to take into account heterogeneous event times and censored observations and hence does not require the specification of a predefined and fixed follow-up time. The CPHM assigns a hazard, i.e. a risk, to every patient for developing a loco-regional recurrence, which can subsequently be used to classify patients into different risk groups for loco-regional failure. The study design is presented in Fig. 1. We investigated four approaches to develop survival models based on the CPHM for the prediction of LRC hazards for patients diagnosed with locally-advanced HNSCC. First, (i) a clinical model was trained on the exploratory cohort and evaluated on the independent validation cohort to provide baseline performance metrics. Moreover, three deep learning based strategies using CNNs were applied: We (ii) trained models completely from scratch, using 3D-CNNs as well as 2D-CNNs, applied (iii) a transfer learning approach leveraging weights of pre-trained 2D-CNN networks, and created (iv) a deep autoencoder and used its bottleneck features in a traditional CPHM.

Prognostic performance was evaluated by two approaches, calculation of the concordance index (C-index) and the ability to stratify patients into two risk groups based on the model predictions. The C-index^37–39^ measures the alignment between the observed times of loco-regional recurrence and the model predictions. It is given on a scale between zero and one with 0.5 indicating no prognostic value of the model. A C-index close to zero represents perfect predictions, since predicted hazards should be lower for patients with a longer recurrence-free time. We emphasise that this is in contrast to the situation of directly predicting event times, where a C-index close to one would be desirable. 95% confidence intervals (CI) for C-indices were computed using the survcomp R package^40,41^ which implements the method proposed by Pencina et al.^42^. Models that did not contain the C-index 0.5 within the 95% CI on the independent validation cohort were considered as successfully validated.

Furthermore, based on the model predictions, patients were assigned to two groups, at low or at high risk for loco-regional recurrence. This stratification was based on the hazard values predicted by the models for every individual patient. The median value of these predictions on the exploratory cohort was used as a cutoff. Patients with a predicted hazard exceeding the cutoff were assigned to the high risk group and the remaining patients with hazards smaller or equal to the cutoff were assigned to the low risk group. To stratify patients of the independent validation cohort, the same cutoff was applied. The difference in LRC between the stratified patient groups was assessed using the log-rank test for the Kaplan–Meier (KM) curves of both risk groups. Significance was established for *p* values below 0.05.

To address the random nature of the CNN training procedure and to leverage the benefits of model ensembles^43^, we repeated model training three times, each time using 10-fold cross-validation (CV) based on the exploratory cohort, stratified by the LRC event status, for a total of 30 CV runs. By applying CV on the exploratory cohort, splits of the samples into training and internal test folds were obtained. Models were built in each CV run using the data of the training fold. Data of the internal test fold was set aside for optional hyperparameter tuning and data of the independent validation cohort was used to measure model performance on previously unseen data.

Since each of the 30 CV runs resulted in a trained model (which we refer to as single model), we created ensemble predictions by averaging of the network outputs, essentially considering the information of multiple models before making a final prediction.

### Image processing

Preprocessing of patient CT scans was carried out using an in-house developed toolkit^44^ (available from https://github.com/oncoray/mirp) by performing (1.) cubic interpolation to isotropic voxel size of $1 \, {\text {mm}}^{3}$, (2.) cropping of the transversal plane to 224 by 224 pixels (with the tumour’s centre of mass as the centre of the cropped slice), (3.) clipping of the intensity range of Hounsfield units (HU) to the range [-200, 200] and (4.) normalisation of pixel values to the interval (0, 1).

Multiple image samples of each patient’s CT scan were extracted and used for model training and prediction. For all 2D-CNN models, we used 7 slices cranial and 8 slices caudal of the slice with the largest tumour area as provided by the segmentation mask, comprising a total of 16 transversal CT slices per patient. For training of the 3D-CNNs we used smaller image regions of the axial plane due to GPU memory limitations. We first extracted a $32 \times 64 \times 64$ ($\hbox {z} \times \hbox {y} \times \hbox {x}$) sized volume centered at the tumour centre of mass. Then, 15 additional random volumes of the same size were extracted for each patient. The volume centres were uniformly sampled from a cubic region of edgelength 32 around the tumour centre of mass. Zero padding was added to all extracted volumes where necessary. For each of the volumes, a prediction was computed. Those were subsequently averaged to obtain a single prediction for each patient.

### Cox proportional hazards model

The traditional CPHM fits the effect of *p*-dimensional covariates ${\varvec{x}}$ on the hazard function *h* via $h(t, {\varvec{x}}) = h_{0}(t) \exp \left( \sum _{j=1}^{p}\beta _{j}x_{j}\right) ,$ with an unspecified baseline hazard function $h_{0}(t)$. We followed Katzman et al.^29^ in extending this to the more general form of $h(t, {\varvec{x}}) = h_{0}(t) \exp \left( \gamma _{\varvec{\beta }}({\varvec{x}})\right)$ with $\varvec{\beta }$ denoting weights learned by a neural network. Log-hazard values $\gamma _{\varvec{\beta }}({\varvec{x}})$ were estimated from CT image samples ${\varvec{x}}$ by minimisation of (a batch approximation of) the negative of the Cox partial log-likelihood function

1 $$\begin{aligned} \ln L = \sum _{i=1}^{n} \delta _{i} \left( \gamma _{\varvec{\beta }} ({\varvec{x}}_{i}) - \ln \left( \sum _{\begin{array}{c} j=1\\ t_{j} \ge t_{i} \end{array}}^{n}\exp (\gamma _{\varvec{\beta }}({\varvec{x}}_{j}))\right) \right) , \end{aligned}$$

letting $\delta _{i}$ denote an event indicator variable that takes on the value 1 if loco-regional tumour recurrence was observed for CT sample *i* and 0 otherwise, and *n* being the total number of available CT samples. Further details on survival analysis and the CPHM are given in “Survival analysis and deep Cox proportional hazards modelling” section of the supplement.

All computations were done using Python 3.6.7 and Keras 2.2.4^45^ with tensorflow (v1.12.0) backend. Our code is publically available from https://github.com/oncoray/cnn-hnscc and experimental outputs can be downloaded from http://doi.org/10.14278/rodare.255.

### Clinical model

To develop the clinical CPHM, we considered the clinical features patient age, gender, cT-stage, cN-stage, UICC-stage, tumour site, p16 status, pathological grading, smoking status, alcohol consumption and primary tumour volume. These features have already been considered in previous studies^7,35^. Tumour site comprised the values oropharynx, hypopharynx, larynx and oral cavity and was one-hot encoded. Volume was computed by summation of tumour segmentation masks and division by a factor of 1000 to obtain units of ${\text {cm}}^{3}$, followed by a (natural) logarithmic transformation.

Imputation of missing values for cN-stage, pathological grading and smoking status (1, 14 and 23 cases, respectively) was performed through selection of the most frequent value in the exploratory cohort. Due to more missing values (58 cases), p16 was converted into the variables ${\text {p16}}_{{\text {unknown}}}$ and p16. The same was done for alcohol consumption for which there were 96 missing cases. cT, cN, UICC and pathological grading stages were converted into the binary categories ${\text {cT}}<4$, ${\text {cN}}<2$, ${\text {UICC}}<4$^7^ and ${\text {pathological grading}}<2$. Patient age and tumour volume were z-score normalised with means and standard deviations obtained from the exploratory cohort. Clinical features prognostic for LRC were selected by applying a forward variable selection CPHM based on the likelihood ratio test (inclusion $\alpha = 0.05$, exclusion $\alpha = 0.1$) using the exploratory cohort. Finally, a CPHM was trained on the exploratory cohort using the selected features and applied to the independent validation cohort.

### Model ensembles

Due to our cross-validation approach (10-fold CV repeated three times), 30 different models were trained in every analysis. By averaging the resulting predicted log-hazard values, one final ensemble prediction for the hazard of loco-regional recurrence was obtained for every patient. On the independent validation cohort, a patient’s ensemble prediction was computed by averaging over all 30 model predictions. For every patient of the exploratory cohort, a training and an internal test ensemble prediction was computed, since they appeared as part of the training folds and as part of the internal test folds. Training ensemble predictions were obtained by computing for every patient an average over all those 27 models for which that patient was part of the training fold. Similarly, internal test ensemble predictions were computed by only using the remaining three models for which the patient belonged to the internal test fold. For ensemble stratification of patients into groups at low and high risk of loco-regional recurrence, the cutoff value was determined as the median value of the training ensemble predictions.

### Training from scratch

Different network architectures of 3D-CNN and 2D-CNN models were trained from scratch. In all trainings we used the AMSGrad version^46^ of the Adam optimiser to estimate model parameters. For the 3D-CNN experiments the same architecture and hyperparameters as given by Hosny et al.^23^ were used with small changes. Due to a different input shape, the first dense layer contained slightly fewer neurons. In the last layer, a single output neuron with $\tanh$ activation was used instead of two neurons with softmax activation which they used for classification purposes. Each model was trained for a fixed number of 200 epochs with a batch size of 24. Neither data augmentation nor callbacks for early stopping or learning rate adjustments were used.

**Figure 2:**
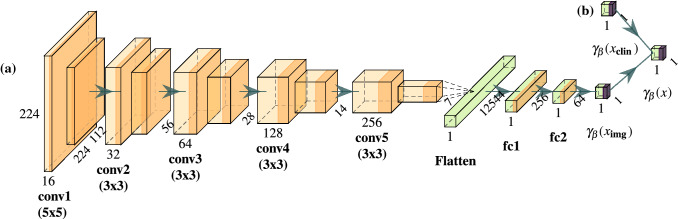
Architecture used when training a 2D-convolutional neural network from scratch. Numbers give shapes of computed feature maps. The network consists of convolutional filters (‘conv’, light orange), with ReLU activation functions (orange). These are followed by a flattening layer and fully-connected dense layers (‘fc’, green). Network output is computed through a $\tanh$ activation (purple). (**a**) This architecture was used when training only on image data. The model output is given by $\gamma _{\varvec{\beta }}({\varvec{x}}_{{\text {img}}})$. (**b**) An additional dense layer was introduced when clinical features were used in addition to image data. The network output in this case is given by $\gamma _{\varvec{\beta }}({\varvec{x}})$.

The 2D-CNN architecture (Fig. 2) was loosely inspired by the VGG architecture^47^. It consisted of five convolution blocks, each containing two convolutional layers with filter size $5 \times 5$ for the first block and $3 \times 3$ for all remaining blocks and ReLU activation functions. No batch normalisation (BN) was used. The second convolutional layer of each block performed downsampling by using a stride of two. The first block comprised of 16 filters. The number of filters was doubled in each subsequent block. A flattening operation and a dropout layer (p=0.3) followed the last convolutional layer, before being connected to two fully-connected dense layers with ReLU activation of sizes 256 and 64, respectively. Dropout with the same probability as above was also applied between those dense layers. Lastly, the model output was given by a single dense neuron with $\tanh$ activation. Training was done for a maximum of 50 epochs with a learning rate of $5\cdot 10^{-5}$ while doing early stopping (patience=10) on the internal test fold as well as reducing learning rates on plateaus (factor=0.5, patience=3, $\min\_\hbox {lr}=10^{-7}$) via the provided Keras callbacks. We also evaluated performance after replacing the final $\tanh$ activation with a linear output, essentially allowing for unrestricted log-hazard ranges. Moreover, the effect of inserting BN layers between convolutions and ReLU activations was assessed.

The effect of combining clinical features and CT samples as two separate inputs to a 2D-CNN was evaluated. First, Spearman correlation coefficients between the 2D-CNN model-output (with BN and $\tanh$ as final activation) and the clinical features were computed. Then, a second input branch, designed to estimate log-hazard values from the clinical features was added to the network architecture as depicted in Fig. 2b). It consisted of a single dense neuron with $\tanh$ activation and with BN. The log-hazard estimates coming from the clinical branch and the image branch were then concatenated and fed through the final output layer consisting again of a single dense neuron with $\tanh$ activation and with BN.

### Transfer learning

We evaluated the capabilities of transfer learning for training 2D-CNNs. The ResNet50^48^, DenseNet201^49^ and InceptionResNetV2 (IRNV2)^50^ architectures with weights pre-trained on the ImageNet dataset were used as foundation models. Their fully connected layers were replaced by a global average pooling layer followed by three dense layers with 128, 32 and one neurons, respectively. The first two dense layers utilised the ReLU activation function and the final layer used the $\tanh$ activation to restrict hazard output to the range $(\exp (-1), \exp (1))$. No BN was applied in the newly added layers.

We ran two experiments per architecture, using the last convolutional layer (denoted “last”) and an earlier layer^51^ (denoted by the name of the layer in the Keras implementation) of the pre-trained networks as foundation for our models. Since those models were trained on RGB images with three input channels, we renormalised each CT slice to the range [0, 255], replicated it to three channels and applied the network preprocessing functions provided by the Keras framework. All layers were fine-tuned simultaneously with a learning rate of $10^{-6}$ for a maximum of 20 epochs while doing early stopping (patience=5) on the internal test fold as well as reducing learning rates on plateaus (factor=0.5, patience=3, $\min\_\hbox {lr}= 10^{-7}$) via the provided Keras callbacks. A batch size of 32 was used and neither data augmentation nor weight regularisation were applied.

### Deep features

**Figure 3:**
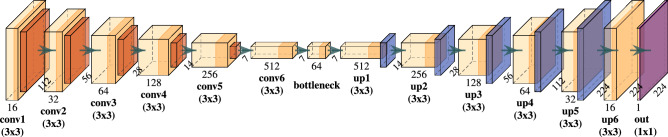
Architecture of the applied autoencoder. Numbers describe the shapes of computed feature maps. Convolutional layers ('conv’) are comprised of convolutional filters (light orange) and Leaky ReLU ($\alpha =0.01$) activation functions (orange). Spatial downsampling is performed using max-pooling layers (red), resulting in a set of bottleneck features. Upsampling operations ('up’, blue), and convolutional layers are then used to reconstruct the input image. A sigmoid activation (purple) is used as model output to match the range of the input data.

Following Wang et al.^34^, we trained a 2D-CNN autoencoder model that learns to reproduce input CT slices as close as possible while passing through a so called bottleneck layer which acts as a means of compression and dimensionality reduction. Successful reconstruction requires capturing of important image characteristics at the bottleneck and we assumed that relevant tumour information was also encoded within those features. The model architecture is provided in Fig. 3 and consisted of an encoder part of six convolutional layers with filter size $3 \times 3$, starting with 16 filters and doubling on each subsequent layer. Leaky ReLU ($\alpha =0.01$) was used as activation. No BN was applied. Between convolutional layers, max-pooling was used to reduce spatial resolution by a factor of two. Finally, a last $3 \times 3$ convolutional layer with 64 filters and the same specification as above was applied to reduce the number of features in the bottleneck representation. The following decoder model was constructed as a mirror image of the encoder using upsampling layers for doubling spatial resolution in each step. The decoder’s last layer was a single $1 \times 1$ convolutional filter with sigmoid activation function to produce outputs with a data range of (0, 1), matching the input image range. Using the binary-crossentropy loss function, we trained the autoencoder for 100 epochs with batches of size 32 using the AMSGrad version of the Adam optimiser with learning rate $10^{-3}$. We used data augmentation by randomly shearing (shear_range = 0.1), zooming (zoom_range = 0.1) and rotating (rotation_range = 45) the input data. We then extracted the bottleneck feature maps of each slice which were of shape $7 \times 7 \times 64$, leading to a reduction to 6.25% of the original image size ($224 \times 224$). Those features were then flattened into a 3136 dimensional vector and a principal component analysis (PCA) was performed using the features of all slices of every patient from the training fold of the CV as a means of dimensionality reduction. Classical CPHMs were subsequently fitted on those training folds using one, two, five and ten PCA features. The learned PCA transformation was then applied to the independent validation cohort features before evaluating the performance of the trained CPHMs on those transformed features. In addition, a Lasso-based CPHM (LCPHM)^52^, that automatically selects relevant features, was fit on the full set of bottleneck features of each training fold without performing a PCA for a maximum of 5000 iterations. The best hyperparameter $\lambda$, which determines the amount of L1 regularisation of the LCPHM, was obtained by another nested CV run on each training fold. This procedure was implemented using the R programming language and the glmnet package^53^.

## Results

### Clinical model

All available clinical features were considered to develop a clinical model for the prediction of LRC hazards. Based on the forward variable selection procedure, only the tumour volume was selected. This univariate CPHM achieved a C-index of 0.39 (95% CI: 0.32-0.45) on the exploratory cohort and a C-index of 0.39 (95% CI: 0.30–0.48) on the independent validation cohort. Stratification of the independent validation cohort into patient groups at low and high risk of loco-regional recurrence based on this clinical model showed a statistical trend approaching significance ($p=0.052$, Supplementary Fig. 1).

### Training from scratch

**Figure 4:**
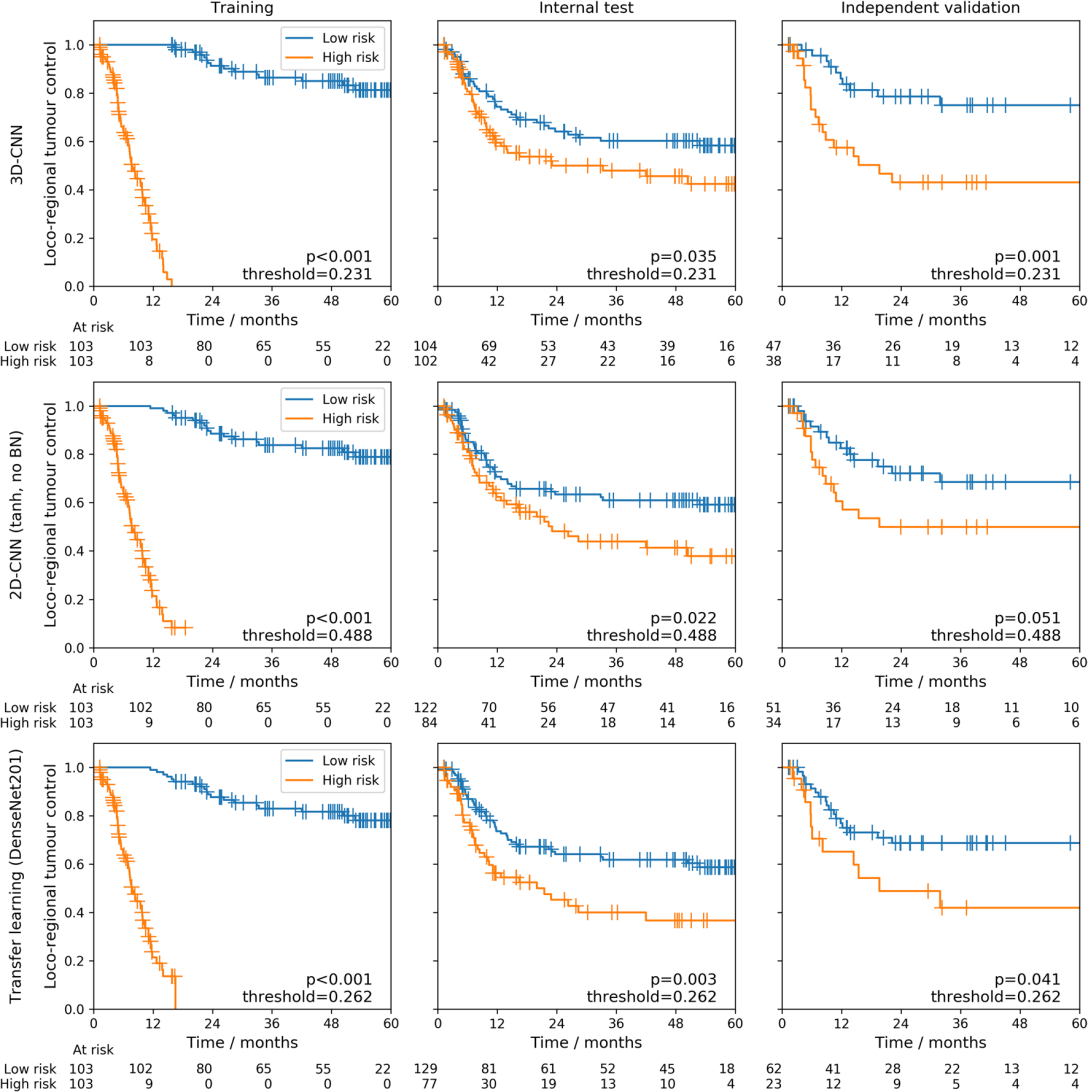
Ensemble Kaplan–Meier curves: Kaplan–Meier curves for patient groups at low risk (blue) and high risk (orange) of loco-regional recurrence for training and internal test folds as well as for the independent validation cohort. The stratification was created using the median of the training ensemble predictions as cutoff. The top row shows the curves obtained from an ensemble of 3D-CNN models trained from scratch based on the architecture of Hosny et al.^23^ with $\tanh$ as final activation. The centre row shows the curves obtained from an ensemble of 2D-CNN models trained from scratch without batch normalisation and $\tanh$ as final activation. The bottom row shows the curves obtained from an ensemble of transfer learning models based on DenseNet201 with the last convolutional layer as foundation.

An ensemble of 3D-CNNs was successfully validated for the prediction of LRC. It achieved a C-index of 0.31 (95%-CI: 0.22-0.39) on the independent validation cohort (Table 2), outperforming the clinical model. Ensembling slightly improved average single model performance (C-index: 0.32, Supplementary Table 1). Moreover, stratification of patients of the independent validation cohort (Fig. 4, top row) into groups at low and high risk of loco-regional recurrence based on the model predictions revealed significant differences in LRC ($p=0.001$). Ensembles of 2D-CNN models trained from scratch were also successfully validated for prognosis of LRC. However, they showed higher C-indices than the 3D model (C-index: 0.38-0.39, Table 2), i.e. a performance comparable to the clinical model. Average single model performance was similar (Supplementary Table 1). All 2D ensemble models led to significant patient stratifications on the independent validation cohort for LRC or showed a statistical trend (Fig. 4, centre row) ($p=0.051$). Table 2 also shows that the inclusion of BN and the choice of final activation did not have a strong impact on performance regarding C-indices or stratification ability of the independent validation cohort. The Spearman correlation coefficient between model predictions and z-score normalised log-tumour volume was moderate across all 30 models (with BN, $\tanh$ as final activation), with average values of 0.30 and 0.36 for the exploratory and independent validation cohort, respectively. Combining imaging data and tumour volume as network input resulted in decreased performance compared to models with only the CT image as input: a C-index of 0.40 (95%-CI: 0.29-0.50) was obtained on the independent validation cohort and model predictions did not result in a statistically significant stratification ($p=0.070$).

**Table 2:** Ensemble training from scratch: C-indices for the endpoint loco-regional control (LRC) are computed by averaging the model predictions of the repeated cross-validation models to build an ensemble model.

Final activation	Batch normalisation	C-index			Log-rank *p* value
		Exploratory cohort		Independent validation cohort	
		Training	Internal test		
3D-CNN					
$\tanh$	Yes	0.02	0.39	**0**.**31**	**0**.**001**
		(0.01–0.03)	(0.33–0.46)	(0.22–0.39)	
2D-CNN					
linear	No	0.02	0.43	0.39	0.039
		(0.01–0.02)	(0.36–0.49)	(0.29–0.49)	
linear	Yes	0.01	0.43	0.38	0.015
		(0.00–0.02)	(0.36–0.49)	(0.27–0.48)	
$\tanh$	No	0.07	0.42	0.38	0.051
		(0.05–0.09)	(0.36–0.48)	(0.28–0.48)	
$\tanh$	Yes	0.01	0.42	0.38	0.015
		(0.01–0.02)	(0.36–0.48)	(0.27–0.48)	
2D-CNN + volume					
$\tanh$	Yes	0.06	0.47	0.40	0.070
		(0.04–0.08)	(0.41–0.53)	(0.29–0.50)	

Values in parenthesis denote 95% confidence intervals. In addition, differences in LRC between Kaplan–Meier curves of the stratified patient groups are assessed by the log-rank test. Best performance is marked in bold.

*C-index* concordance index, $\tanh$, hyperbolic tangent.

### Transfer learning

For transfer learning, the ensemble of DenseNet201 models in combination with its last convolutional layer as the foundation was successfully validated for prognosis of LRC and achieved the best C-index of 0.37 (95%-CI: 0.27-0.47) on the independent validation cohort (Table 3), which was slightly better than the clinical model. Compared to average single model peformance (C-index: 0.41, Supplementary Table 2) this was an improvement of 0.04. Moreover, a statistically significant stratification into low and high risk groups of loco-regional recurrence was achieved by this ensemble for the independent validation cohort (Fig. 4, bottom row) ($p=0.041$). Using the last convolutional layer as foundation, ensembles of ResNet50 or IRNV2 models were not able to successfully stratify patients of the independent validation cohort. Layers different from the last convolutional layer of the pre-trained models as input for the newly added dense layers resulted in slightly worse C-indices in all cases.

**Table 3:** Ensemble of transfer learning models: C-indices for the endpoint loco-regional control (LRC) are computed by averaging the model predictions of the repeated cross-validation models to build an ensemble model.

Architecture	Layer name	C-index			Log-rank *p* value
		Exploratory cohort		Independent validation cohort	
		Training	Internal test		
ResNet50	last	0.06	0.37	0.39	0.17
		(0.04–0.07)	(0.31–0.42)	(0.30–0.48)	
ResNet50	activation_37	0.14	0.39	0.41	0.15
		(0.11–0.17)	(0.33–0.44)	(0.31–0.51)	
DenseNet201	last	0.05	0.39	**0**.**37**	0.041
		(0.04–0.06)	(0.33–0.45)	(0.27–0.47)	
DenseNet201	conv4_block48	0.12	0.43	0.43	0.032
		(0.10–0.15)	(0.37–0.50)	(0.33–0.53)	
IRNV2	last	0.08	0.38	0.41	0.25
		(0.06–0.10)	(0.32–0.44)	(0.31–0.52)	
IRNV2	block17_10_ac	0.26	0.41	0.42	**0**.**023**
		(0.22–0.31)	(0.36–0.47)	(0.32–0.53)	

Values in parenthesis denote 95% confidence intervals. In addition, differences in LRC between Kaplan–Meier curves of the stratified patient groups are assessed by the log-rank test. Best performance is marked in bold.

*C-index* concordance index,* IRNV2* inceptionResNetV2.

Boxplots showing the variability of ensemble predictions for patients of the independent validation cohort are provided in Supplementary Figs. 2, 3 and 4 for the ensemble of 3D-CNN models, 2D-CNN models and DenseNet201 models, respectively.

### Deep features

The prognostic performance of classical CPHMs using bottleneck features of autoencoder models as covariates are given in Table 4. Model performance was inferior to the clinical model in all scenarios and none of the models achieved a statistically significant stratification of the independent validation cohort into low and high risk groups. The best C-index on the independent validation cohort was 0.42 (95%-CI: 0.32–0.53), obtained by the LCPHM ensemble. The ensemble model improved the C-index on the independent validation cohort by 0.03 compared to the average single model C-index (Supplementary Table  3). The amount of the full variance of the data captured by the PCA features is provided in Supplementary Table  4.

**Table 4:** Ensemble of autoencoder models: C-indices for the endpoint loco-regional control (LRC) are computed by averaging the model predictions of the repeated cross-validation models to build an ensemble model.

Feature selection + ML algorithm	C-index			Log-rank *p* value
	Exploratory cohort		Independent validation cohort	
	Training	Internal test		
- + LCPHM	0.01	0.50	**0**.**42**	**0**.**19**
	(0.00–0.01)	(0.43–0.57)	(0.32–0.53)	
PCA(1) + CPHM	0.49	0.53	0.54	0.63
	(0.42–0.56)	(0.47–0.60)	(0.42–0.66)	
PCA(2) + CPHM	0.47	0.51	0.53	**0**.**19**
	(0.40–0.54)	(0.44–0.58)	(0.42–0.64)	
PCA(5) + CPHM	0.44	0.50	0.50	0.72
	(0.37–0.50)	(0.44–0.57)	(0.41–0.60)	
PCA(10) + CPHM	0.35	0.42	0.43	0.40
	(0.29–0.40)	(0.36–0.48)	(0.33–0.53)	

Values in parenthesis denote 95% confidence intervals. In addition, differences in LRC between Kaplan–Meier curves of the stratified patient groups are assessed by the log-rank test. Best performance is marked in bold.

* C-index* concordance index,* ML* machine learning,* CPHM* Cox proportional hazards model,* LCPHM* Lasso-Cox proportional hazards model,* PCA* principal component analysis.

## Discussion

We investigated deep learning methods in a survival analysis setting for the endpoint LRC, based on treatment-planning CT images of locally advanced HNSCC patients treated with primary radiochemotherapy. Best performance and successful validation was achieved by an ensemble of 3D-CNNs with a C-index of 0.31 on the independent validation cohort. Patient risk groups defined by the model predictions showed significant differences in LRC ($p=0.001$). Ensembles of different 2D-CNN approaches performed similar to a clinical CPHM based on the tumour volume (independent validation C-index of 0.39). Compared to using only a single trained model instance, our analysis revealed benefits in using model ensembles for final predictions, which is in line with the reasoning of Dietterich^43^.

Overall, reported performances for 2D-CNNs were comparable to results previously published from our group by Leger et al.^17^. They evaluated multiple combinations of feature selection algorithms and classical machine learning models based on handcrafted radiomics features on the same dataset. An average independent validation C-index over all combinations of 0.62 was achieved (which corresponds to a C-index of 0.38 in our context, as explained in the “Methods” section). Similarly, Haarburger et al.^28^ reported C-indices between 0.585 and 0.623 using a CNN based CPHM on a CT imaging dataset of lung cancer patients. However, they suggested to reformulate the regression problem as a classification task. This was due to their GPU memory limitations which did not allow large enough batch sizes for good approximations of the partial log-likelihood function of the CPHM. We did not observe problems with small batch sizes (see Supplementary Table 5), but investigated their approach in an additional analysis: We evaluated ensemble model performance of 2D-CNNs using a cutoff of 24 months for LRC. Samples of 72 patients previously used in our analysis had to be discarded due to censoring before the cutoff value, clearly demonstrating the downside of binarising the time-to-event variable. We achieved an area under the receiver operating characteristic (AUROC) curve of 0.60 on the independent validation cohort which is comparable to the reported AUROC values of 0.598 and 0.636^28^.

We also provided empirical evidence (Supplementary Table 5) that approximation of the Cox partial log likelihood by batches does not seem to be problematic since models were able to learn well on the training set regardless of small (32) or large (256) batch sizes. However, we did not observe additional benefits for increased batch sizes. Moreover, we found that adding more samples of a single patient for training and inference did not further improve 2D-CNN results (Supplementary Table 5).

Using deep autoencoder features turned out to be the least effective approach among our investigations, since it never achieved statistically significant patient stratifications and showed the worst C-indices on the independent validation cohort. This might be due to having used a too large compression in our network design of the bottleneck features or having chosen a too small amount of PCA features. This is indicated by the performance improvements when switching from five to 10 PCA features, as well as by results reported by Wang et al.^34^. There, using 16 autoencoder features (of a different network architecture and without PCA), C-indices of 0.713 and 0.694 on two cohorts diagnosed with high-grade serous ovarian cancer were reported.

Most CNN models struggled with overfitting, as can be seen in the large discrepancies of C-indices between training and internal test/independent validation (Tables 2, 3), which is also reflected in the large separation of KM curves between low and high risk groups in the training column of Fig. 4. Adding multiple regularisation approaches such as L1 and L2 weight regularisation, increased dropout rates and data augmentation to the fitting procedure of our 2D-CNN models trained from scratch, we observed indeed drops in training performance for most approaches but without improvements on the independent validation, Supplementary Table 6. Similar observations were made for the 3D-CNN models. There, we employed a 3D data augmentation strategy using code from https://github.com/MIC-DKFZ/batchgenerators which included elastic deformations (deformation scale (0, 0.25)), random rotations in the range of [− 15, 15] degrees for each of the three spatial axes, random rescaling in the range of (0.75, 1.25), mirroring, random brightness multiplications in the range (0.7, 1.5), gaussian noise additions with noise variance parameter set to (0, 0.05) and per channel gamma transformations using a gamma range parameter of (0.5, 2). However, ensemble results were similar (independent validation C-index 0.30, log-rank $p=0.003$) to the results obtained without using data augmentation. Throughout our experiments, we also observed C-index performance on the internal test folds of the CV to have much higher variance compared to results of the training folds and the independent validation. We attribute this to the small sample sizes of about 20 patients in the internal test folds during the 10-fold CVs. Ensemble predictions for the internal test patients were also consistently worse than the ensemble predictions for the patients of the independent validation cohort, which might be due to the smaller number of models used for building the ensembles (only three models for the former but 30 for the latter) and inherent statistical differences between patients of the exploratory and the independent validation cohort for, e.g. tumour volume or tumour site.

The performance benefits observed for 3D-CNNs may have multiple causes. Firstly, those models allowed to incorporate potentially relevant spatial (three dimensional) context around the tumour during training. In contrast, 2D-CNN models by design were not capable of exploiting this additional information during training, but only during inference, by considering predictions of multiple patient slices. Secondly, the input image size differed between 2D and 3D-CNN models. While 2D-CNNs analysed the full axial plane, 3D-CNN models processed only a relatively small axial area close to the tumour which might have allowed them to learn more relevant tumour features and not to get distracted by possibly uninformative image regions. Thirdly, the model’s architecture and hyperparameters differed between 2D and 3D-CNN models which could have influenced the observed performance differences.

Our analysis contains some limitations: Our data set, even though competitive in size in the field of medical imaging, might be too small to obtain better results. For 2D-CNN models, even transfer learning was not able to circumvent this limitation, which might also be due to the large translational gap that exists between natural RGB images and CT scans. Federated learning^54,55^ seems to be a promising way to tackle the small sample size problem of medical imaging. This includes setting up infrastructures to allow to collaboratively train models on data of multiple institutions without violating data-privacy regulations. Also, exploring generative adversarial networks for enhancing dataset sizes through simultaneous generation of synthetic image samples and plausible time-to-event labels^56,57^ might provide a potentially interesting task. However, for HNSCC, treatment-planning CT scans may simply not contain much more predictive information to achieve better performance, no matter the deep learning approach, model architecture or hyperparameters. As previously indicated^3,9,58^, considering additional imaging during the course of treatment or additional imaging modalities such as MRI or PET may offer improved predictive potential. Another limitation of our analysis concerns the Cox partial log-likelihood function, as given by equation (1), which does not account for ties in the data. This can very well occur if multiple samples of the same patient are present in a single training batch. Therefore, we plan on using e.g. Efrons correction method^59^ in future analysis but refrained from that in our current experiments in order to avoid introduction of additional complexity in the loss function. Instead, we experimented with using slight random perturbations on the observed event times to avoid exact matches. We did, however, not observe noteworthy changes in model performance (see first row of Supplementary Table 6). An alternative to the CPHM is the combination of deep learning with accelerated failure time models, as demonstrated by Chapfuwa et al.^57^ on clinical data. Due to their fully-parametric nature, direct prediction of event times becomes easier and non-monotonic hazard functions can be modelled.

Deep learning approaches on treatment-planning CT images can be useful building blocks on the way to achieve the goal of personalisation of radiotherapy. They may be extended using additional information, e.g. from tumour histology or molecular samples. Nevertheless, deep learning approaches should not be considered the universal remedy since they also bring with them some drawbacks compared to simpler models. Those include increased computational complexity and difficulties in understanding the image-based causes of their predictions, leading to decreased model interpretability.

In this study, we implemented CNNs for the prediction of LRC after primary radiochemotherapy of locally advanced HNSCC based on CT imaging. An ensemble of 3D-CNN models was successfully validated and showed an improved performance compared to 2D-CNN approaches and a clinical model. Risk groups defined on these models differed significantly in LRC. In the future, we aim to assess robustness and translational ability of our trained models by applying them to data of the prospective HNPrädBio trial of the DKTK-ROG as another independent validation (NCT02059668)^60^.

## Supplementary information

Supplementary Information.

## Data Availability

The datasets used and analysed during the current study are available from the corresponding author on reasonable request. Experimental output and trained models are accessible from http://doi.org/10.14278/rodare.255. Python and R code of our analyses is available from https://github.com/oncoray/cnn-hnscc.
