# Supplementary material for: 2D and 3D convolutional neural networks for outcome modelling of locally advanced head and neck squamous cell carcinoma
Source: Sci Rep. 2020 Sep 24;10:15625. doi: 10.1038/s41598-020-70542-9 (PMC7518264; doi:10.1038/s41598-020-70542-9)
Supplement: Supplementary file 1 — Supplementary Information. [file 41598_2020_70542_MOESM1_ESM.pdf]

Supplement to: 2D and 3D convolutional neural networks for outcome modelling of locally advanced head and neck squamous cell carcinoma

Sebastian Starke[1,2,3,*], Stefan Leger[2,3,4], Alex Zwanenburg[2,3,4], Karoline Leger[2,3,4,5], Fabian Lohaus[2,3,4,5], Annett Linge[2,3,4,5], Andreas Schreiber[6], Goda Kalinauskaite[7,8], Inge Tinhofer[7,8], Nika Guberina[9,10], Maja Guberina[9,10], Panagiotis Balermpas[11,12], Jens von der Grün[11,12], Ute Ganswindt[13,14,15,16], Claus Belka[13,14,15], Jan C. Peeken[13,17,18], Stephanie E. Combs[13,17,18], Simon Böke[19,20], Daniel Zips[19,20], Christian Richter[2,3,5,21], Esther G.C. Troost[2,3,4,5,21], Mechthild Krause[2,3,4,5,21], Michael Baumann[2,3,4,5,21,22], and Steffen Löck[2,3,5]

[*]s.starke@hzdr.de
[1]Helmholtz-Zentrum Dresden - Rossendorf, Department of Information Services and Computing, Dresden, Germany
[2]OncoRay - National Center for Radiation Research in Oncology, Faculty of Medicine and University Hospital Carl Gustav Carus, Technische Universität Dresden, Helmholtz-Zentrum Dresden - Rossendorf, Dresden, Germany
[3]German Cancer Research Center (DKFZ), Heidelberg and German Cancer Consortium (DKTK) partner site Dresden, Germany
[4]National Center for Tumor Diseases (NCT), Partner Site Dresden, Germany: German Cancer Research Center (DKFZ), Heidelberg, Germany; Faculty of Medicine and University Hospital Carl Gustav Carus, Technische Universität Dresden, Dresden, Germany, and; Helmholtz Association / Helmholtz-Zentrum Dresden - Rossendorf (HZDR), Dresden, Germany
[5]Department of Radiotherapy and Radiation Oncology, Faculty of Medicine and University Hospital Carl Gustav Carus, Technische Universität Dresden, Dresden, Germany
[6]Department of Radiotherapy, Hospital Dresden-Friedrichstadt, Dresden, Germany
[7]German Cancer Research Center (DKFZ), Heidelberg and German Cancer Consortium (DKTK) partner site Berlin, Germany
[8]Department of Radiooncology and Radiotherapy, Charité University Hospital, Berlin, Germany
[9]German Cancer Research Center (DKFZ), Heidelberg and German Cancer Consortium (DKTK) partner site Essen, Germany
[10]Department of Radiotherapy, Medical Faculty, University of Duisburg-Essen, Essen, Germany
[11]German Cancer Research Center (DKFZ), Heidelberg and German Cancer Consortium (DKTK) partner site Frankfurt, Germany
[12]Department of Radiotherapy and Oncology, Goethe-University Frankfurt, Germany
[13]German Cancer Research Center (DKFZ), Heidelberg and German Cancer Consortium (DKTK) partner site Munich, Germany
[14]Department of Radiation Oncology, Ludwig-Maximilians-Universität, Munich, Germany
[15]Clinical Cooperation Group, Personalized Radiotherapy in Head and Neck Cancer, Helmholtz Zentrum, Munich, Germany
[16]Department of Radiation Oncology, Medical University of Innsbruck, Anichstraße 35, A-6020, Innsbruck, Austria
[17]Department of Radiation Oncology, Technische Universität München, Germany
[18]Institute of Radiation Medicine (IRM), Helmholtz Zentrum München, Neuherberg, Germany
[19]German Cancer Research Center (DKFZ), Heidelberg and German Cancer Consortium (DKTK) partner site Tübingen, Germany
[20]Department of Radiation Oncology, Faculty of Medicine and University Hospital Tübingen, Eberhard Karls Universität Tübingen, Germany
[21]Helmholtz-Zentrum Dresden - Rossendorf, Institute of Radiooncology – OncoRay, Dresden, Germany
[22]German Cancer Research Center (DKFZ), Heidelberg, Germany

ABSTRACT

For treatment individualisation of patients with locally advanced head and neck squamous cell carcinoma (HNSCC) treated with primary radiochemotherapy, we explored the capabilities of different deep learning approaches for predicting loco-regional tumour control (LRC) from treatment-planning computed tomography images. Based on multicentre cohorts for exploration (206 patients) and independent validation (85 patients), multiple deep learning strategies including training of 3D- and 2D-convolutional neural networks (CNN) from scratch, transfer learning and extraction of deep autoencoder features were assessed and compared to a clinical model. Analyses were based on Cox proportional hazards regression and model performances were assessed by the concordance index (C-index) and the model's ability to stratify patients based on predicted hazards of LRC. Among all models, an ensemble of 3D-CNNs achieved the best performance (C-index 0.31) with a significant association to LRC on the independent validation cohort. It performed better than the clinical model including the tumour volume (C-index 0.39). Significant differences in LRC were observed between patient groups at low or high risk of tumour recurrence as predicted by the model ($p = 0.001$). This 3D-CNN ensemble will be further evaluated in a currently ongoing prospective validation study once follow-up is complete.

1 Survival analysis and deep Cox proportional hazards modelling

Survival analysis aims at finding stochastic models for a patient's survival time, which is assumed to be a random variable T. Its survival function is denoted by $S(t) = P(T > t)$. The hazard rate is introduced via $h(t) = f(t)/S(t)$ where f denotes the probability density function of T. The connection between survival and hazard function is established via

$$S(t) = \exp\left(-\int_0^t h(\tau)\mathrm{d}\tau\right). \tag{1}$$

The Cox proportional hazards model (CPHM) in its traditional form is used to estimate the influence of covariates $x = (x_1, \ldots, x_p)$ on the hazard rate via

$$h(t,x) = h_0(t) \cdot \exp(\beta_1 x_1 + \ldots + \beta_p x_p) = h_0(t) \cdot \exp\left(\beta^T x\right). \tag{2}$$

Katzman et al.[1] proposed to change the model function to the more general form of

$$h(t,x) = h_0(t) \cdot \exp\left(\gamma_\beta(x)\right), \tag{3}$$

where $\gamma_\beta(x)$ is an arbitrary function parametrised by β, e.g. a neural network. This has the benefit of capturing nonlinear interaction terms between model covariates which is not the case in the traditional form (2). Model parameters β can then be estimated by optimisation of the Cox partial log-likelihood:

$$\ln L = \sum_{i=1}^{n} l(x_i, y_i) = \sum_{i=1}^{n} l(x_i, (\delta_i, t_i)) = \sum_{i=1}^{n} \delta_i \left(\gamma_\beta(x_i) - \ln\left(\sum_{\substack{j=1 \\ t_j \geq t_i}}^{n} \exp(\gamma_\beta(x_j))\right)\right). \tag{4}$$

The modified model formulation gives a straightforward algorithmic idea for the application of deep learning procedures, which estimate parameters using gradient descent based optimisation methods. Due to the batch-wise training of CNNs, only small chunks of b samples of the full dataset ($b \ll n$) are used to compute an approximation $\sum_{i=1}^{b} l(x_i, y_i) \approx \sum_{i=1}^{n} l(x_i, y_i)$ of the full loss function for carrying out a parameter update step. This was shown to work well in practice for many traditional loss functions. However, to model time-to-event survival data according to (4), one has to note that not only the outer sum is an approximation but also the inner sum running over j can only be computed for the samples in a batch and not on the full sample, leading to the log-likelihood approximation

$$\ln L_b = \sum_{i=1}^{b} \delta_i \left(\gamma_\beta(x_i) - \ln\left(\sum_{\substack{j=1 \\ t_j \geq t_i}}^{b} \exp(\gamma_\beta(x_j))\right)\right) \tag{5}$$

used for computing parameter estimates. In order to evaluate the effect of this batch approximation, we performed different experiments using a 2D-CNN model which was trained from scratch. This consisted of five convolutional blocks with two convolutional layers each, followed by a flattening operation and three dense layers with 256, 64 and 1 units, respectively. The filter size was 5x5 for the first convolutional block and 3x3 for the remaining blocks. The second layer within each block

performed spatial downsampling using a stride of two. ReLU activation was used in all convolutional filters and all dense dense layers except for the last one, which used tanh as final activation. Batch normalisation was not used in the model. We incrementally increased batch sizes b, leaving all other hyperparameters unchanged. In order to avoid increased loss values and potentially numeric instabilities due to larger batches, we changed the summation in (5) into an average, effectively computing $\frac{1}{b}\ln L_b$. At the same time, we evaluated whether adding more slices per patient to training and inference procedures can improve prognostic performance. As Table 5 suggests, increasing batch sizes did not improve results for the independent validation cohort. In general, only training performance was affected. Since, in theory, larger batches achieve better approximations to the true log-likelihood, we expected improved performance when increasing batch sizes. However, our results showed decreased training and nearly unaltered independent validation cohort performance. Moreover, adding more slices for each patient did not further improve prediction and even reduced the fraction of significant stratifications for all investigated batch sizes up to 128.

2 Regularisation

Since the presented results for 2D-CNNs clearly show signs of overfitting, we evaluated whether standard procedures known as regularisation methods can have a positive impact on performance. We investigated the following methods: increased dropout rates, weight regularisation penalising the L1 and L2 norms of learned model parameters and data augmentation. We built upon the same 2D-CNN architecture that we trained from scratch and which was described in the first section. We used the same hyperparameters as a baseline setup (batch size of 32, no batch normalisation and tanh final activation) and adjusted only the regularisation parameters in the following ways:

- Dropout: increased dropout rate between dense layers from 0.3 on the baseline setup to 0.5
- Perturbation of event-time labels when replicating the patients event time to individual slices by a random value drawn from the uniform(-0.1, 0.1) distribution
- Penalisation of model weights via L1 and L2 norm with penalty factor of 10^{-5} as additional term to the loss function
- Data augmentation applied randomly to input images during training, using
  - shear range = 0.2
  - zoom range = 0.2
  - rotation range = 30
  - fill mode = nearest

The results are summarised in Table 6. No major improvements compared to the independent validation cohort concordance index (C-index) of 0.38 of the baseline setup were observed.

3 Ensemble variations

For each of the ensemble models for which we have shown Kaplan-Meier curves, we provide a plot containing boxplots of model predictions for all patients of the independent validation cohort. This illustrates the variability of predictions across models for each patient, as well as the capability of the model to predict higher risk values for patients with shorter event times compared to patients with longer event times.

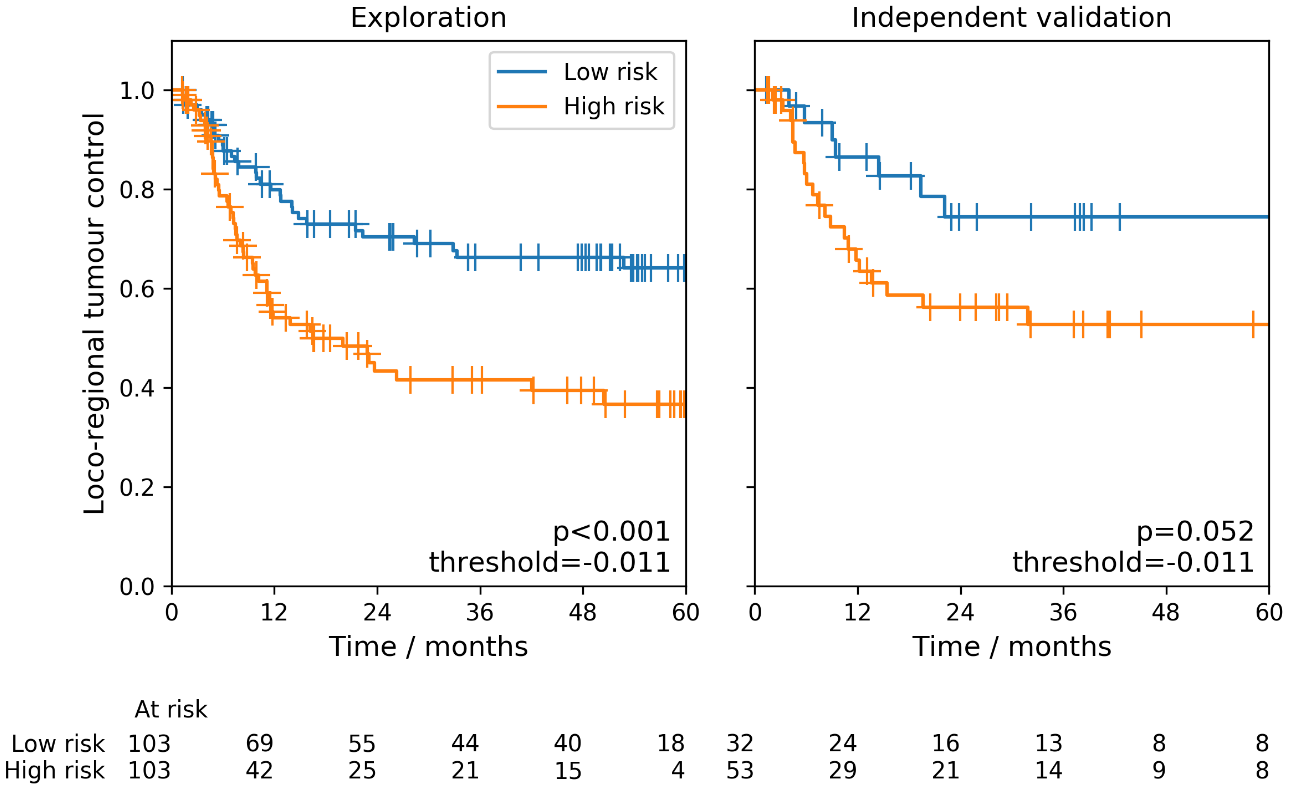

Figure 1. Clinical model: Kaplan Meier curves for low risk (blue) and high risk groups (orange) for the exploratory and independent validation cohort. The stratification was created using the median of the exploratory cohort predictions as cutoff.

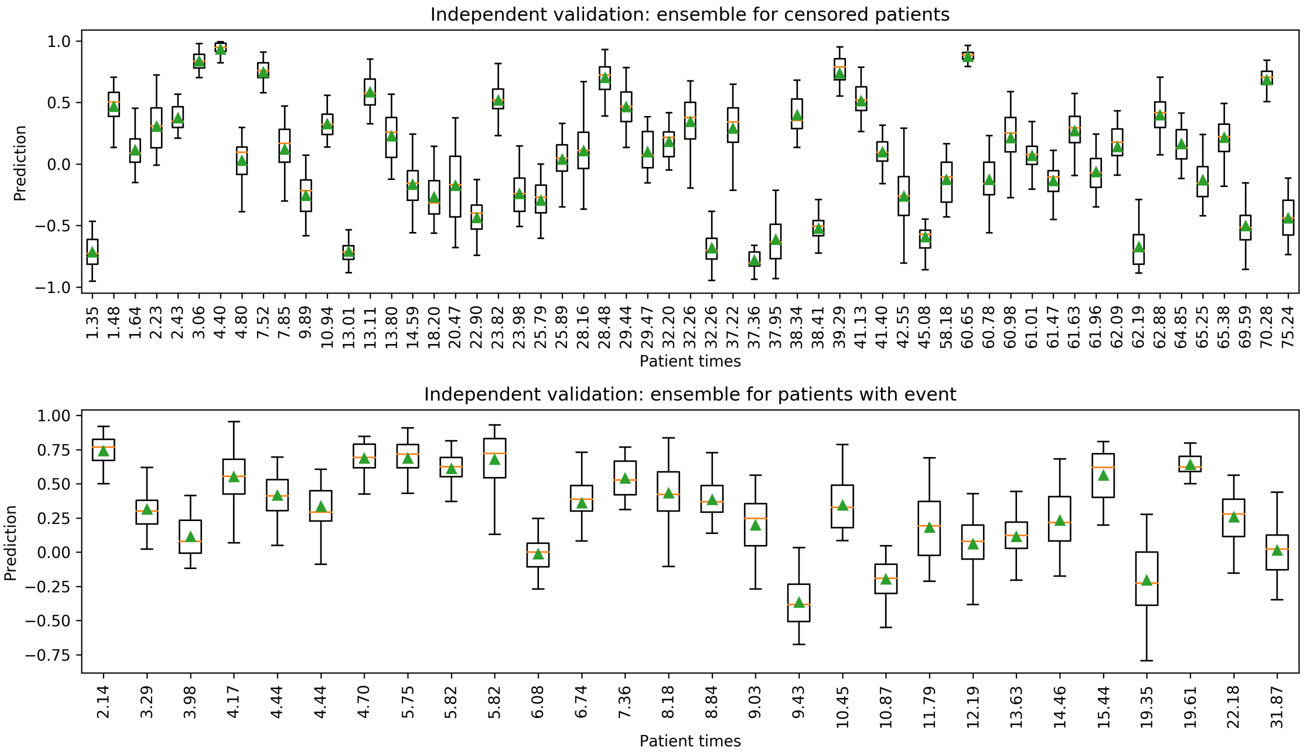

Figure 2. **Ensemble training from scratch (3D-CNN):** Boxplots showing the variability of model predictions used in the ensemble for all patients of the independent validation cohort using models based on the architecture of Hosny et al.[2] with tanh as final activation. Boxplots are given separately for patients with and without observed event. Patients are ordered by increasing event times. Means are given in green, median values in yellow.

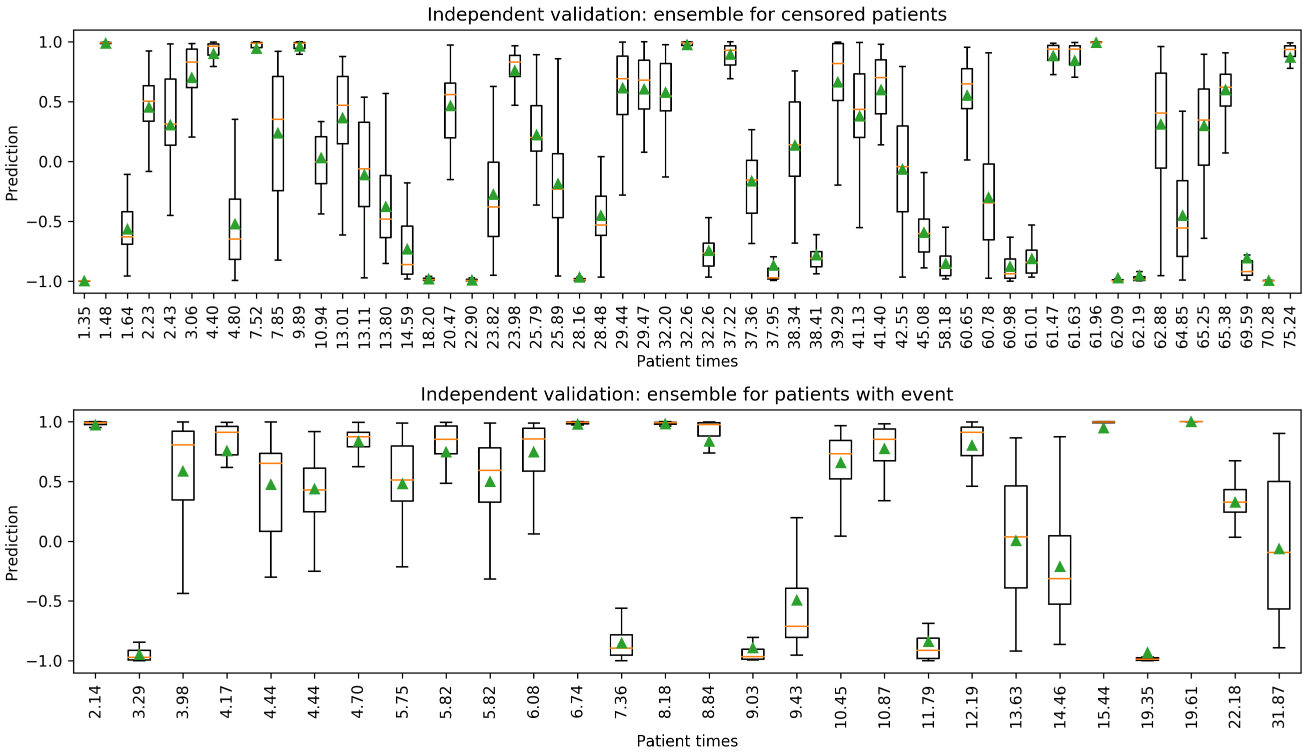

Figure 3. Ensemble training from scratch (2D-CNN): Boxplots showing the variability of model predictions used in the ensemble for all patients of the independent validation cohort using models trained from scratch with tanh final activation and no batch normalisation. Boxplots are given separately for patients with and without observed event. Patients are ordered by increasing event times. Means are given in green, median values in yellow.

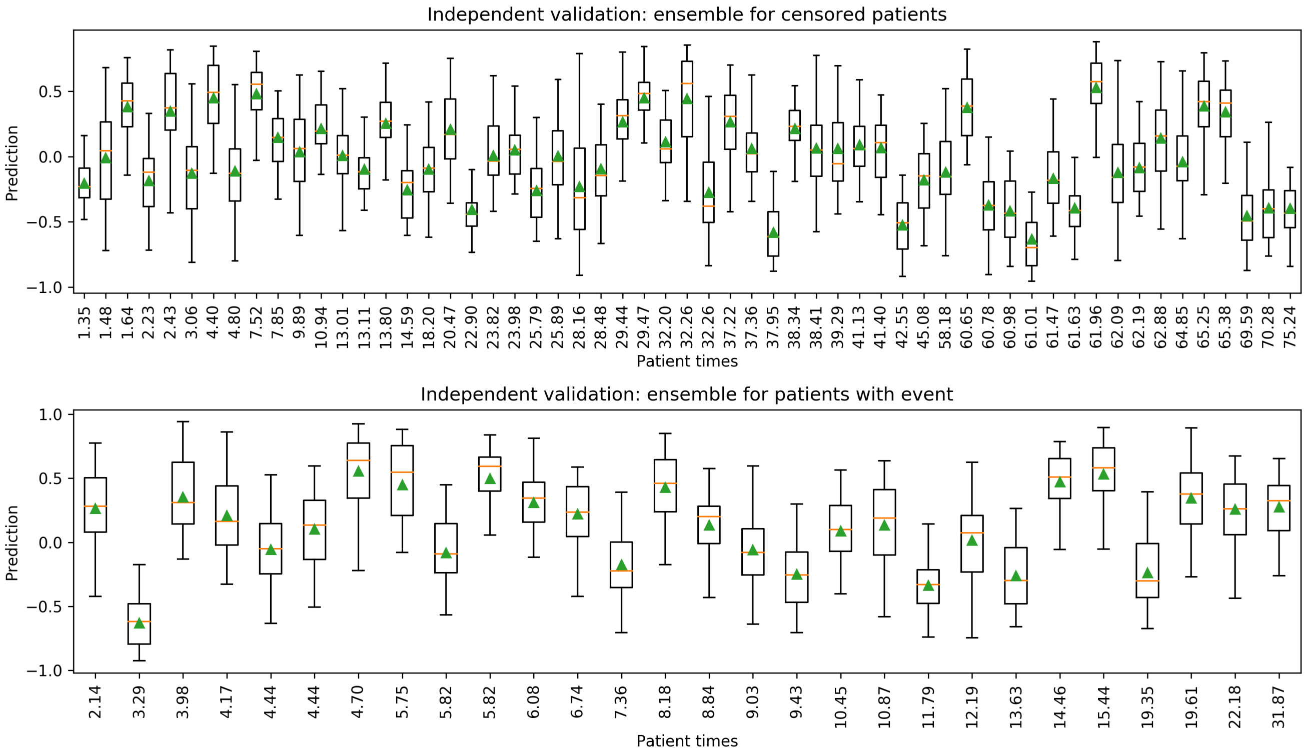

Figure 4. Ensemble of transfer learning models: Boxplots showing the variability of model predictions used in the ensemble for all patients of the independent validation cohort using transfer learning models based on DenseNet201 with last convolutional layer as foundation. Boxplots are given separately for patients with and without observed event. Patients are ordered by increasing event times. Means are given in green, median values in yellow.

Table 1. Training from scratch: all results are averaged over three repetitions of 10-fold cross-validation. Values in parenthesis denote minimum and maximum, best performance is marked in bold.

Final activation	Batch normalisation	C-index			Fraction of Log-rank p-value < 0.05
		Exploratory cohort		Independent validation cohort	
		Training mean (min - max)	Internal test mean (min - max)	mean (min - max)	
3D-CNN					
tanh	yes	0.03 (0.02 - 0.04)	0.39 (0.18 - 0.74)	**0.32 (0.27 - 0.37)**	**22/30**
2D-CNN					
linear	no	0.02 (0.01 - 0.04)	0.43 (0.21 - 0.68)	0.40 (0.37 - 0.42)	8 / 30
linear	yes	0.02 (0.01 - 0.03)	0.42 (0.24 - 0.59)	0.39 (0.35 - 0.44)	13 / 30
tanh	no	0.09 (0.05 - 0.14)	0.43 (0.23 - 0.67)	0.38 (0.35 - 0.44)	18 / 30
tanh	yes	0.02 (0.01 - 0.03)	0.43 (0.24 - 0.66)	0.40 (0.33 - 0.44)	8 / 30
2D-CNN + volume					
tanh	yes	0.12 (0.02 - 0.57)	0.47 (0.28 - 0.73)	0.44 (0.32 - 0.61)	11/30

Abbreviations: C-index, concordance index; 2D-CNN, two dimensional convolutional neural network; 3D-CNN, three dimensional convolutional neural network; tanh, hyperbolic tangent

Table 2. Transfer learning models: the models were pre-trained on the ImageNet dataset and fine-tuned on the considered CT dataset. All results are averaged over three repetitions of 10-fold cross-validation. Values in parenthesis denote minimum and maximum, best performance is marked in bold.

Architecture	Layer name	C-index			Fraction of Log-rank p-value < 0.05
		Exploratory cohort		Independent validation cohort	
		Training mean (min - max)	Internal test mean (min - max)	mean (min - max)	
ResNet50	last	0.08 (0.04 - 0.15)	0.38 (0.17 - 0.63)	**0.41 (0.34 - 0.51)**	5 / 30
ResNet50	activation_37	0.18 (0.08 - 0.30)	0.38 (0.20 - 0.61)	0.43 (0.38 - 0.47)	3 / 30
DenseNet201	last	0.08 (0.03 - 0.18)	0.40 (0.21 - 0.62)	**0.41 (0.33 - 0.51)**	**15 / 30**
DenseNet201	conv4_block48	0.21 (0.07 - 0.40)	0.44 (0.22 - 0.70)	0.44 (0.39 - 0.48)	5 / 30
IRNV2	last	0.13 (0.04 - 0.24)	0.40 (0.16 - 0.60)	0.43 (0.33 - 0.50)	7 / 30
IRNV2	block17_10_ac	0.28 (0.22 - 0.39)	0.40 (0.19 - 0.66)	0.43 (0.38 - 0.47)	11 / 30

Abbreviations: C-index, concordance index; IRNV2, InceptionResNetV2

Table 3. Autoencoder models: results in terms of C-indices using transformations of the $7 \times 7 \times 64$ bottleneck features of autoencoder models in combination with traditional CPHMs. All results are averages over three repetitions of 10-fold cross-validation. The fraction of models with a statistically significant stratification of the independent validation cohort is provided in the fifth column. Values in parenthesis denote minimum and maximum, best performance is marked in bold.

Feature selection + ML algorithm	C-index			Fraction of Log-rank p-value < 0.05
	Exploratory cohort		Independent validation cohort	
	Training	Internal test		
	mean (min - max)	mean (min - max)	mean (min - max)	
- + LCPHM	0.01 (1e-4 - 0.08)	0.48 (0.25 - 0.77)	0.45 (0.40 - 0.54)	0 / 30
PCA(1) + CPHM	0.49 (0.46 - 0.52)	0.52 (0.31 - 0.78)	0.53 (0.42 - 0.55)	0 / 30
PCA(2) + CPHM	0.47 (0.44 - 0.49)	0.49 (0.16 - 0.80)	0.52 (0.47 - 0.55)	0 / 30
PCA(5) + CPHM	0.44 (0.41 - 0.47)	0.50 (0.33 - 0.73)	0.50 (0.48 - 0.54)	0 / 30
PCA(10) + CPHM	0.35 (0.32 - 0.40)	0.42 (0.22 - 0.64)	**0.43 (0.40 - 0.46)**	0 / 30

Abbreviations: C-index, concordance index; ML, machine learning; CPHM, Cox proportional hazards model; LCPHM, Lasso-Cox proportional hazards model; PCA, principal component analysis

Table 4. Autoencoder models: amount of explained variance in the encoding features of the training folds when using PCA. All results are averages over three repetitions of 10-fold cross-validation. Values in parenthesis denote minimum and maximum.

PCA dimensions	Fraction of explained variance mean (min - max)
1	0.17 (0.14 - 0.43)
2	0.31 (0.27 - 0.74)
5	0.50 (0.45 - 0.96)
10	0.62 (0.57 - 0.99)

Abbreviations: PCA, principal component analysis

Table 5. Training from scratch (2D-CNN): predictive performance for different batch sizes and numbers of slices per patient. All results are averaged over three repetitions of 10-fold cross-validation. Values in parenthesis denote minimum and maximum, best performance is marked in bold.

Batch size	Number of input slices	C-index			Fraction of Log-rank p-value < 0.05
		Exploratory cohort		Independent validation cohort	
		Training mean (min - max)	Internal test mean (min - max)	mean (min - max)	
32	16	0.09 (0.05 - 0.13)	0.44 (0.19 - 0.66)	**0.39 (0.35 - 0.43)**	**14 / 30**
32	32	0.06 (0.05 - 0.09)	0.43 (0.29 - 0.68)	0.40 (0.37 - 0.43)	3 / 30
32	48	0.05 (0.04 - 0.06)	0.45 (0.25 - 0.72)	0.41 (0.37 - 0.45)	1 / 30
64	16	0.14 (0.07 - 0.24)	0.44 (0.21 - 0.64)	0.40 (0.36 - 0.44)	11 / 30
64	32	0.08 (0.06 - 0.12)	0.45 (0.28 - 0.69)	**0.39 (0.36 - 0.43)**	7 / 30
64	48	0.06 (0.05 - 0.08)	0.47 (0.29 - 0.72)	0.41 (0.36 - 0.44)	2 / 30
128	16	0.22 (0.08 - 0.38)	0.44 (0.20 - 0.67)	0.41 (0.36 - 0.47)	11 / 30
128	32	0.11 (0.06 - 0.19)	0.44 (0.27 - 0.60)	0.40 (0.35 - 0.44)	5 / 30
128	48	0.08 (0.05 - 0.13)	0.45 (0.28 - 0.74)	0.41 (0.36 - 0.44)	7 / 30
256	16	0.30 (0.18 - 0.42)	0.45 (0.24 - 0.70)	0.42 (0.39 - 0.46)	4 / 30
256	32	0.20 (0.09 - 0.34)	0.45 (0.21 - 0.68)	0.40 (0.38 - 0.44)	5 / 30
256	48	0.13 (0.08 - 0.22)	0.44 (0.24 - 0.77)	0.41 (0.37 - 0.44)	4 / 30

Abbreviations: C-index, concordance index

Table 6. Training from scratch (2D-CNN): effect of regularisation on predictive model performance. All results are averages over three repetitions of 10-fold cross-validation. Values in parenthesis denote minimum and maximum, best performance is marked in bold.

Regularisation	C-index			Fraction of Log-rank p-value < 0.05
	Exploratory cohort		Independent validation cohort	
	Training mean (min - max)	Internal test mean (min - max)	mean (min - max)	
Baseline (dropout=0.3)	0.09 (0.05 - 0.14)	0.43 (0.23 - 0.67)	**0.38 (0.35 - 0.44)**	**18/30**
time perturbation	0.09 (0.06 - 0.15)	0.44 (0.16 - 0.65)	0.39 (0.35 - 0.43)	10 / 30
L1 + L2	0.08 (0.05 - 0.15)	0.45 (0.29 - 0.62)	0.39 (0.35 - 0.45)	15 / 30
Dropout=0.5	0.10 (0.06 - 0.22)	0.44 (0.20 - 0.65)	0.40 (0.35 - 0.44)	14 / 30
Data augmentation, no dropout	0.23 (0.11 - 0.31)	0.44 (0.19 - 0.70)	0.41 (0.36 - 0.45)	6 / 30
Data augmentation	0.29 (0.14 - 0.38)	0.41 (0.25 - 0.71)	0.42 (0.37 - 0.45)	5 / 30
Data augmentation + dropout=0.5	0.33 (0.17 - 0.40)	0.44 (0.23 - 0.64)	0.42 (0.39 - 0.45)	2 / 30
Data augmentation + dropout=0.5 + L1 + L2	0.34 (0.24 - 0.39)	0.43 (0.23 - 0.64)	0.42 (0.35 - 0.45)	8 / 30

Abbreviations: C-index, concordance index

References

1. Katzman, J. L. *et al.* DeepSurv: Personalized treatment recommender system using a Cox proportional hazards deep neural network (2018). arXiv:1606.00931v3.

2. Hosny, A. *et al.* Deep learning for lung cancer prognostication: A retrospective multi-cohort radiomics study. *PLoS Medicine* **15**, 1–25 (2018).
